# Total Patient Delay: A Comparison of Patient and Clinician/Health System Delays in the Diagnosis of Progressive Supranuclear Palsy and Corticobasal Syndrome

**DOI:** 10.1002/mdc3.13990

**Published:** 2024-02-18

**Authors:** Diane M.A. Swallow, Peter Murchie, Carl E. Counsell

**Affiliations:** ^1^ Institute of Applied Health Sciences University of Aberdeen Aberdeen United Kingdom

**Keywords:** progressive supranuclear palsy, corticobasal degeneration, Parkinson's disease, misdiagnosis, delayed diagnosis, appraisal delay, illness delay, behavioral delay, total patient delay

## Abstract

**Background:**

Early diagnosis in progressive supranuclear palsy (PSP) and corticobasal syndrome (CBS) is important for clinical care and key to developing successful disease‐modifying agents. The patient‐dependent phases of decision‐making made before contact with a healthcare professional have been inadequately studied.

**Objectives:**

To evaluate the patient‐dependent phases of decision‐making from symptom onset, comparing this to clinician and/or health system delays within the overall diagnostic pathway.

**Methods:**

Using the Anderson General Model of Total Patient Delay and a mixed‐methods approach in participants with PSP/CBS and their caregivers recruited to the Scottish PSP and CBS cohort, we quantified and evaluated the determinants of “appraisal”, “illness,” and “behavioral” delay, comparing this to the clinician and/or health system delays (“treatment” delay) within the overall time from symptom onset to diagnosis.

**Results:**

The time from index symptom onset to diagnosis was 3.26 (interquartile range [IQR] = 2.42, 4.75) years in PSP and 2.58 (IQR = 1.69, 4.08) years in CBS. Patient appraisal delay was 24 (IQR = 6, 60) weeks in PSP and 8 (IQR = 5, 24) weeks in CBS, illness delay 0 (IQR = −14, 0) weeks in PSP and 0 (IQR = −4, 0) weeks in CBS, with little perceived behavioral delay. Determinants of delay included the non‐specificity of symptoms, normalization of symptoms within the context of age or normal physiological variability, and the extent of insight into new somatic symptoms.

**Conclusions:**

Although patient appraisal delay contributes to overall diagnostic delay in PSP/CBS, the greater proportion of overall diagnostic delay arises after contact with a healthcare professional (treatment delay).

## Introduction

Progressive supranuclear palsy (PSP) and corticobasal syndrome (CBS) are rare, neurodegenerative tauopathies with some overlap in clinical features. Misdiagnosis and delayed diagnosis are common, but few studies have systematically described the evolution of the diagnostic process from symptom onset to death to identify variables amenable to intervention to improve diagnostic timeliness and accuracy. Early diagnosis is important for clinical care, ensuring individuals can access vital support and benefits, plan for the future, and avoid incorrect diagnosis, inappropriate treatment, and miscommunication of prognosis. It is also a key consideration for advancing the translation of future therapeutic approaches into successful disease‐modifying agents^1^; enabling individuals with PSP/CBS to be identified early in their disease course while they still meet eligibility criteria, such as independent ambulation, for disease‐modifying clinical trials and before the development of severe neurodegeneration decreases any potential benefit from disease‐modifying agents. Although diagnostic and exclusionary biomarkers may eventually facilitate the diagnostic process, they can only be used once an individual has sought healthcare input and is at least suspected to have a relevant clinical syndrome or specific diagnosis.

**FIG. 1:**
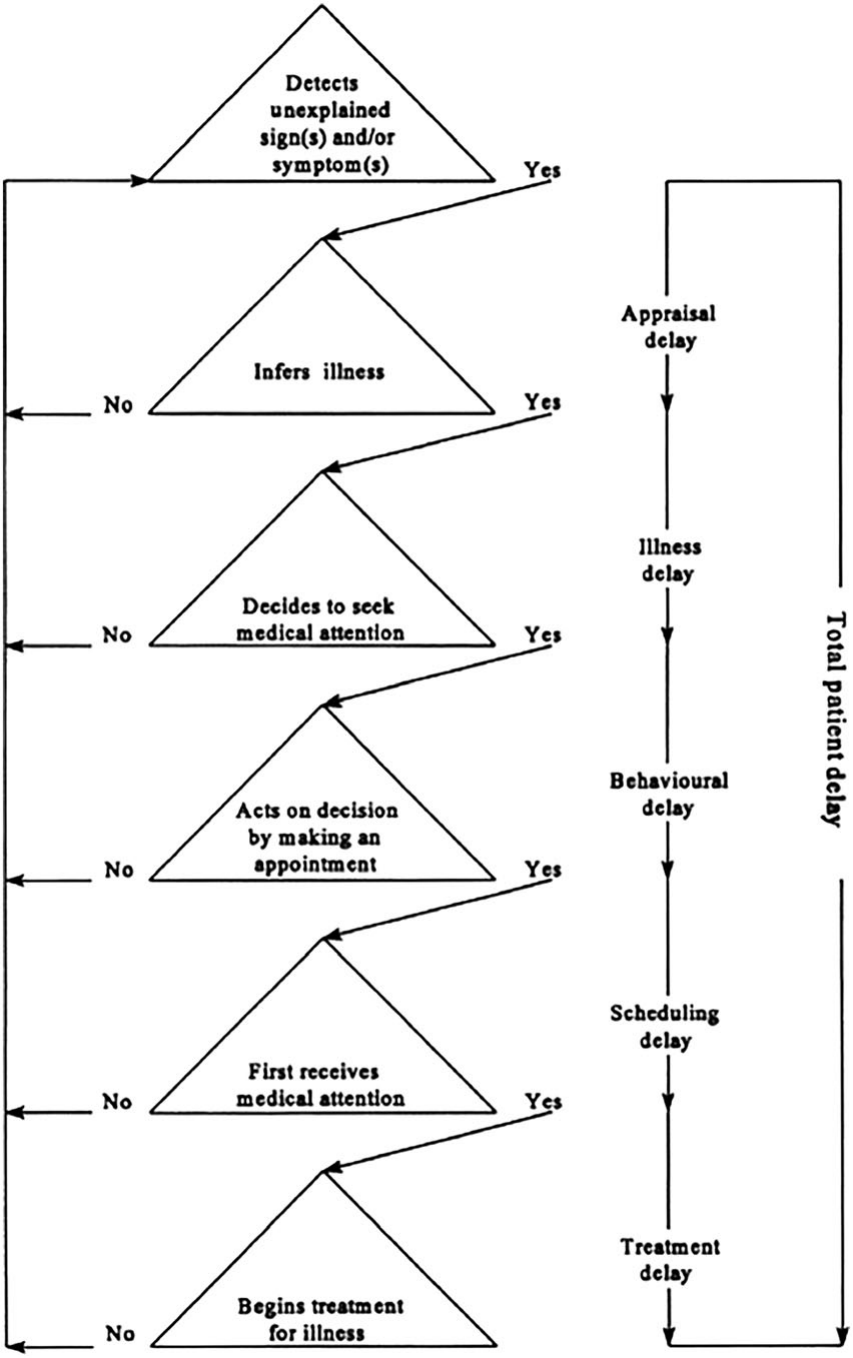
The General Model of Total Patient Delay as proposed by Anderson et al (1995).

The Anderson General Model of Total Patient Delay (Fig. 1), a theoretical framework that conceptualizes delay intervals occurring between phases of decision‐making from symptom detection to treatment onset, is often used in cancer diagnostics research.^2^ This model has multiple patient‐dependent decision‐making phases made before contact with a healthcare professional, including “appraisal delay” (the time period from symptom detection to the evaluation of a symptom as a sign of illness), “illness delay” (the time from evaluation of a symptom as a sign of illness to the decision to seek professional medical care), and “behavioral delay” (the time between deciding medical professional input is required and acting on this decision). These components of overall diagnostic delay cannot be retrospectively extracted from the medical record and therefore, have not, to our knowledge, been explored in the existing PSP/CBS literature. As such, neither the relative quantitative contribution of patient's health‐related behaviors to overall diagnostic delay in PSP/CBS, nor the personal, inter‐personal, or environmental determinants influencing these phases of decision‐making, are well understood.

We, therefore, used a cross‐sectional mixed‐method analysis, using participants with PSP/CBS and their caregivers recruited to the national Scottish PSP and CBS cohort to: (1) quantify and evaluate the personal, inter‐personal or environmental determinants of the patient‐dependent phases of decision‐making from index symptom onset to contact with a healthcare professional using an established framework (Anderson General Model of Total Patient Delay); (2) compare the duration of patient‐dependent and healthcare system‐dependent phases of diagnostic delay (“treatment delay”) within the overall time to diagnosis (symptom onset to diagnosis).

## Methods

### Study Population: Scottish PSP and CBS Cohort

The Scottish PSP and CBS cohort comprises prevalent patients with PSP/CBS, their principal caregivers (related or non‐related), and age‐, sex‐, and disease duration‐ matched Parkinson's disease (PD) controls, recruited from across Scotland from January 2018 to July 2019. Referrer (hospital clinician, PD nurse specialist, or patient self‐referral) diagnoses were confirmed by clinical examination by a neurology trainee (D.M.A.S.), with videoed clinical assessments also reviewed by a consultant neurologist with a specialist interest in movement disorders (C.E.C.), according to consensus diagnostic criteria,^3^, ^4^, ^5^ or where this was impossible, by case note review. PSP/CBS participants were invited to consent (assent sought from caregivers where those with PSP/CBS did not have capacity to provide written consent) to baseline and 6‐month clinician and patient completed clinical assessments (detailed in Table S1) encompassing a variety of motor, cognitive, functional disability, quality of life, and neuropsychiatric clinical domains, as well as questionnaires on diagnostic and care pathways (Table S2). Participants could also consent to linkage to electronic health records (both patient/caregiver), structural and functional magnetic resonance imaging (MRI), and post‐mortem confirmation of diagnosis (PSP/CBS participants only). All caregivers completed proxy assessments of PSP/CBS participant abilities, as well as questionnaires relating to their own health status and carer input (Table S3), whereas participants with PD undertook motor and cognitive assessments (Table S4) and MRI.

### Current Analysis

Data from questionnaires, completed independently by PSP/CBS patients and their caregivers, were entered verbatim into an electronic database for the current analysis. Index symptoms were defined as a participants' first self‐reported symptom/s. All PSP/CBS participants were asked to provide the month/year (all dates assigned the first of each month) of: (1) index symptom onset; (2) their first review by their general practitioner (GP); (3) their first referral to secondary care; and (4) their final diagnosis of PSP/CBS. PSP/CBS participants were also asked to estimate the time interval (weeks/months/years) between index symptom onset and their first evaluation of an index symptom/s as serious or a sign of illness (used to calculate average appraisal delay [median, interquartile range (IQR)]), the time interval between index symptom onset and their first decision to seek the input of a healthcare professional (used to calculate average illness delay [median, IQR]), and to indicate if they had actively delayed seeking medical attention having deemed this necessary (behavioral delay). Treatment delay in the Anderson model is defined as the time between an individual's first appointment with a health care professional and the onset of treatment. In this analysis, we used the date of diagnosis as a surrogate for treatment commencement. Caregivers were similarly invited to report the time interval between index symptom onset and the decision of their relative to seek the input of a healthcare professional and their relative's date of diagnosis. Both patients and caregivers were asked to report the speciality of their initial secondary care referral, the speciality making their final diagnosis of PSP/CBS and any preceding alternative diagnoses. Where participants responded in units only (eg, weeks), unit midpoints were used as estimates for the purposes of analysis. For example, a time interval of “months” was estimated to be 6 months, whereas “weeks” was estimated as 2 weeks.

Qualitative data were analyzed using thematic analysis. Coding procedures were undertaken after data from all participants had been collected and entered into the study database. All responses made by patients and caregivers were read through in one session with brief initial impressions and identification of key points identified. The text responses to each individual question were subsequently thoroughly and systematically reviewed to identify discrete ideas, perceptions, or behaviors. Codes were further created or refined as each new individual's data was systematically reviewed and were named using a newly generated, rather than a pre‐specified, naming convention. Similar concepts were then grouped into higher order categories or themes.^6^

## Results

### Sample Demographics

**FIG. 2:**
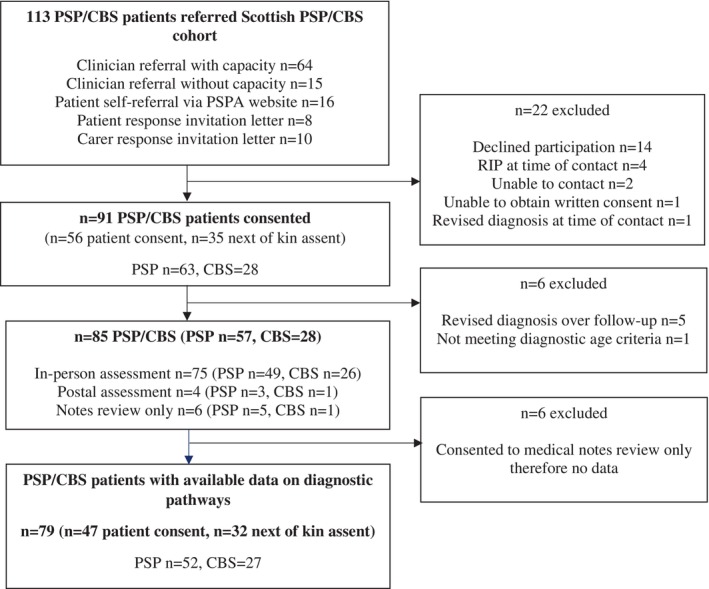
Progressive supranuclear palsy (PSP) and corticobasal syndrome (CBS) Scottish cohort study PSP/CBS participant profile.

Of 113 referrals made to the Scottish PSP and CBS cohort, 22 were excluded, leaving 91 PSP/CBS patients (PSP, n = 63; CBS, n = 28) who consented (n = 56 patient consent, n = 35 next of kin assent) (Fig. 2). In addition to PSP/CBS participants, 81 caregivers and 83 PD age‐, sex‐, and disease duration matched controls were also recruited. The baseline characteristics of PSP/CBS participants recruited to the study is shown in Table 1. PSP/CBS patients were 70 years on average, with no clear sex differences. Most were white (over 90%), married (over 60%), and living at home (over 85%) with nominated caregivers primarily their spouse or partner (70.4%). Because of subsequent exclusions (eg, diagnostic revisions) or consent preferences (eg, consent to notes review only), detailed in Figure 2, data on diagnostic pathways for the current analysis was available in 52 PSP patients (n = 32 patient consent, n = 20 next of kin assent) and 41 PSP caregivers, and 27 CBS patients (n = 15 patient consent, n = 12 next of kin assent) and 17 CBS caregivers.

**TABLE 1:** Baseline demographics of PSP and CBS patients and caregivers recruited to the Scottish PSP and CBS cohort

	PSP/CBS patients, n = 91		Caregivers, n = 81
	PSP, n = 63	CBS, n = 28	
Sex (male)	29 (46.0)	16 (57.1)	35 (43.2)
Age at assessment (mean, SD)	70.3 (6.8)	70.1 (8.4)	62.7 (12.8)
Marital status			
Married	42 (66.7)	17 (60.7)	64 (79.0)
Single/widowed/divorced	19 (30.2)	10 (35.7)	13 (16.0)
Missing	2 (3.2)	1 (3.6)	4 (5.0)
Education status (completed)			–
Primary	63 (100)	28 (100)	
Secondary	45 (71.4)	19 (67.9)	
Tertiary	11 (17.4)	6 (21.4)	
Residence at baseline			–
Home	56 (88.9)	25 (89.3)	
Home of relative	1 (1.6)	0 (0.0)	
Sheltered accommodation	3 (4.8)	1 (3.6)	
Nursing home	3 (4.8)	2 (7.1)	
Carer relationship to patient	–	–	
Spouse or partner			57 (70.4)
Sibling			2 (2.5)
Daughter			12 (14.8)
Son			5 (6.2)
Friend			1 (1.2)
Professional carer			1 (1.2)
Other			3 (3.7)

*Note*: Numbers are frequency (percentage) unless otherwise stated.

Abbreviations: CBS, corticobasal syndrome; SD, standard deviation; PSP, progressive supranuclear palsy.

### Index Symptoms

Including both PSP/CBS patient responses and proxy caregiver responses where no patient response was provided (predominantly because of incapacity), 27.7% PSP patients and 42.9% of CBS patients experienced multiple symptoms at onset. In PSP, the most prevalent index symptoms were falls (36.2%), mobility difficulties (23.4%), and visual disturbance (19.1%), whereas in CBS coordination difficulties (38.1%), altered cognition (23.8%), and tremor (19.0%) were most prevalent. Patient and caregiver reporting of index symptoms were largely consistent except for speech/swallowing and behavioral symptoms in PSP/CBS, which were more commonly reported by caregivers than patients (Table 2).

**TABLE 2:** Index symptoms self‐reported by PSP and CBS patients and their caregivers

Index symptoms	PSP					CBS				
	Patient n = 52	Caregiver n = 41	Patient or proxy caregiver^a^ n = 47	Matched patient‐caregiver responses n = 23		Patient n = 27	Caregiver n = 17	Patient or proxy caregiver^a^ n = 21	Matched patient‐proxy caregiver responses n = 10	
				Patient	Caregiver				Patient	Caregiver
Missing incapacity	19 (36.5) 12 (23.1)	4 (9.8)	–	–	–	11 (40.7) 9 (33.3)	0 (0.0)	–	–	–
Multiple	8 (15.4)	16 (39.0)	13 (27.7)	6 (26.1)	11 (47.8)	5 (18.5)	9 (52.9)	9 (42.9)	4 (40.0)	5 (50.0)
Mobility	9 (17.3)	8 (19.5)	11 (23.4)	8 (34.8)	5 (21.7)	3 (11.1)	3 (17.6)	3 (14.3)	1 (10.0)	3 (30.0)
Balance (not including falls)	5 (9.6)	4 (9.8)	6 (12.8)	4 (17.4)	3 (13.0)	1 (3.7)	1 (5.9)	2 (9.5)	1 (10.0)	0 (0.0)
Falls	9 (17.3)	15 (36.6)	17 (36.2)	6 (26.1)	8 (34.8)	2 (7.4)	2 (11.8)	3 (14.3)	1 (10.0)	2 (20.0)
Coordination	1 (1.9)	1 (2.4)	1 (2.1)	1 (4.3)	0 (0.0)	7 (25.9)	7 (41.2)	8 (38.1)	5 (50.0)	6 (60.0)
Vision	6 (11.5)	5 (12.2)	9 (19.1)	5 (21.7)	2 (8.7)	0 (0.0)	0 (0.0)	0 (0.0)	0 (0.0)	0 (0.0)
Speech or swallowing	4 (7.7)	9 (22.0)	5 (10.6)	2 (8.7)	6 (26.1)	1 (3.7)	2 (11.8)	2 (9.5)	0 (0.0)	1 (0.0)
Tremor	1 (1.9)	0 (0.0)	1 (2.1)	0 (0.0)	0 (0.0)	2 (7.4)	2 (11.8)	4 (19.0)	1 (10.0)	0 (0.0)
Slowness	1 (1.9)	1 (2.4)	1 (2.1)	1 (4.3)	1 (4.3)	1 (3.7)	0 (0.0)	1 (4.8)	1 (10.0)	0 (0.0)
Stiffness	0 (0.0)	0 (0.0)	0 (0.0)	0 (0.0)	0 (0.0)	2 (7.4)	0 (0.0)	2 (9.5)	2 (20.0)	0 (0.0)
Cognitive	2 (3.8)	4 (9.8)	3 (6.4)	2 (8.7)	3 (13.0)	3 (11.1)	5 (29.4)	5 (23.8)	3 (30.0)	3 (30.0)
Behavior	2 (3.8)	8 (19.5)	5 (10.6)	1 (4.3)	5 (21.7)	0 (0.0)	1 (5.9)	0 (0.0)	0 (0.0)	1 (10.0)
Sensory, weakness, or alien limb	0 (0.0)	0 (0.0)	0 (0.0)	0 (0.0)	0 (0.0)	1 (3.7)	3 (17.6)	3 (14.3)	1 (10.0)	1 (10.0)
Other	1 (1.9)^b^	2 (4.9)^c^, ^d^	1 (2.1)^c^	1 (4.3)^b^	2 (8.7)^b^, ^d^	1 (3.7)^e^	1 (5.9)^b^	2 (9.5)^d^, ^e^	1 (10.0)^e^	1 (10.0)^b^

Abbreviations: CBS, corticobasal syndrome; PSP, progressive supranuclear palsy.

^a^ Patient responses plus carer responses only where no patient response was provided (because of incapacity or simply missing).

^b^ Sialorrhea.

^c^ Restless legs.

^d^ Change in handwriting.

^e^ Dizziness.

### Patient Phases of Decision‐Making

#### Appraisal Delay

The average patient reported appraisal delay was three times longer in PSP than CBS (24 weeks vs. 8 weeks) (Table 3). A quarter (n = 13, 25.0%) of PSP patients inferred illness at symptom onset, whereas the remainder with available data (n = 22, 42.3%) initially attributed their symptoms to other causes. A third (n = 9, 33.3%) of those with CBS initially attributed their index symptoms to illness (Table S5).

**TABLE 3:** Total patient delay (appraisal, illness, behavioral, and treatment delay) in PSP and CBS

Time intervals (weeks unless stated otherwise)	PSP, n = 52	CBS, n = 27
Appraisal delay	24 (IQR = 6–60) [n = 29]^a^	8 (IQR = 5–24) [n = 15]^b^
Illness delay	0 (IQR = −14 to 0) [n = 29]^c^	0 (IQR = −4 to 0) [n = 13]^d^
Behavioral delay	n = 5 reported delay, none quantified	n = 1 reported delay (4 weeks)
Treatment delay		
First GP attendance to first secondary care referral	13 (IQR = 0–26) [n = 21]^e^	11 (IQR = 3–15) [n = 8]^f^
First secondary care referral to diagnosis of PSP/CBS (years)	1.92 (IQR = 1.08–3.73) [n = 22]^g^	0.91 (IQR = 0.44–1.75) [n = 9]^h^
To final diagnosis (years)	3.26 (IQR = 2.42–4.75) [n = 49]^i^	2.58 (IQR = 1.69–4.08) [n = 26]^j^

*Note*: Appraisal delay: n = 1 PSP and n = 2 CBS responded in units only (months), estimated to be 6 months for the purpose of analysis. Illness delay: 2 PSP patients units only. Treatment delay including first GP attendance to first secondary care referral: n = 2 PSP provided the year only (assigned mid‐point June for analysis). Index symptom to first GP review n = 9 PSP units only (years), 6 giving approximation within year (eg, “early”).

Abbreviations: CBS, corticobasal syndrome; IQR, interquartile range; GP, general practitioner; PSP, progressive supranuclear palsy.

^a^ No data in n = 12 without capacity, n = 3 unable to recall interval, n = 2 who did not infer illness until diagnosis, n = 6 missing data.

^b^ No data in n = 10 without capacity, n = 2 missing data.

^c^ No data in n = 14 without capacity, n = 9 missing data.

^d^ Excluding n = 10 without capacity, n = 1 unable to recall, n = 1 referred by another secondary care physician, n = 2 missing data.

^e^ n = 1 missing date of first GP attendance only, n = 10 missing date of first referral secondary care only, n = 19 missing both (including n = 13 without capacity), and excluding n = 1 where date of referral preceded date of initial GP attendance.

^f^ n = 2 missing date of first GP attendance only, n = 4 date of first secondary care referral, and n = 13 both (including n = 9 without capacity).

^g^ n = 29 missing date of GP referral, n = 1 excluded as date of diagnosis preceded date of GP referral.

^h^ n = 17 missing date of GP referral, n = 1 excluded as date of diagnosis preceded date of GP referral.

^i^ n = 2 missing date of index symptom, n = 1 date of index symptom excluded as >10 year from first GP review.

^j^ n = 1 missing date of index symptom.

The determinants that influenced PSP and CBS patient's appraisal of their symptoms and their initial or eventual inference of illness are outlined in detail in Table S5. The type or nature of individual symptoms, conceptual beliefs that symptoms were because of a serious specific diagnosis or illness, and the impact or threat associated with symptoms (eg, falls injury) accounted for an immediate appraisal of perceived symptoms as serious or because of illness. Some people did not immediately attribute symptoms to illness, but evaluated them as to be expected within physiological variation, normalized them within a wider physical or psychological context (for example arising because of injury or anxiety), or decided to monitor them over time. However, if the symptoms became worse, persisted, or started to interfere with daily activities, they were reinterpreted over time as being abnormal. Abnormal symptoms or signs were, however, not always recognized by individuals as such, and social factors including the actions of both family members and GPs were, for some, instrumental in influencing health seeking behavior and perception of illness. Several individuals with both PSP and CBS, for example, reported they had only recognized their symptoms as potentially serious based on the actions of their GP, after having sought help.

#### Illness Delay

Having appraised perceived symptoms to be because of illness in 24 weeks (PSP) and 8 weeks (CBS) on average, individuals with PSP and CBS decided to see their GP 24 (IQR = 2–52) weeks and 6 (IQR = 1–12) weeks after index symptom onset, respectively (10 PSP and six CBS patients sought the input of their GP before evaluating their symptoms as an indicator of illness, whereas one PSP patient was hospitalized before seeking the input of their GP). PSP caregivers similarly reported that PSP patients had sought the input of their GP at ~24 (IQR = 4–52) weeks after symptom onset, whereas CBS caregivers reported a slightly longer average interval of 10 (IQR = 3.5–27) weeks. The average illness delay or time from evaluating symptoms as a sign of illness to the decision to seek professional medical care, in both PSP and CSB was, therefore, 0 weeks (Table 3).

As in appraisal delay, an individuals' decision to seek the input of a healthcare professional was influenced by an evaluation of the nature and consequences of symptoms experienced, including their persistence and impact on functional ability, an inability to manage or self‐monitor such symptoms alone (requiring, for example, the use of emergency services or believing that medication changes were required) or having attributed symptoms to specific causes. The wishes, recommendations, or perceived concerns of family members and other social contacts as reasons to seek help from their GP were particularly influential at this phase of decision‐making. Emotion, in particular anxiety, also influenced the help‐seeking process (Table S5).

#### Behavioral Delay

When individuals with PSP and CBS were asked directly whether, after having decided to see their GP, they delayed or held off making an appointment, most reported no delay. Five PSP and one CBS patient indicated a delay in making an appointment to see their GP having deemed this necessary. The reasons for delay appear to relate to outcome expectancy; ranging from a belief that a simple non‐urgent intervention was required (medication change) to a degree of denial or an awareness of the possibly of receiving a life‐changing diagnosis and a hope that symptoms would resolve. A self‐defined threshold of symptom frequency or severity justifying GP help‐seeking also appears to influence this phase of decision‐making (Table S5).

### Treatment Delay

After reaching primary care, the average reported duration between first GP attendance to first referral to secondary care was 13 weeks in PSP and 11 weeks in CBS (Table 3). Approximately half of initial GP referrals were to specialities with movement disorder experience (neurology or Care of the Elderly [COTE]) with the remainder sent to a range of other medical and surgical specialities (Table 4). Eight PSP patients, unprompted, described the input of multiple secondary care specialities (n = 6, two specialities; n = 2, three specialities). Three PSP patients solely commented on the perceived reluctance of their GP to take their symptoms seriously or refer to secondary care. Three patients sought private healthcare input (referrals to neurology and orthopedics). Fewer CBS patients received input from multiple secondary care specialities, with just one patient reporting their review by neurology occurred after an initial referral to stroke. Across the health boards of Scotland over half (55.8%) of PSP patients recalled being given at least one alternative diagnosis, and approximately a fifth (21.2%) multiple diagnoses, either in primary or secondary care, before their diagnosis of PSP. A higher proportion (70.4%) of CBS patients reported at least one alternative diagnosis, but fewer (7.4%) multiple preceding diagnoses. Although parkinsonism, in particular PD, was a common misdiagnosis, patients with both PSP and CBS received a breadth of other explanations for their symptoms (Table 4). Most PSP/CBS patients received their final diagnosis by a neurologist or COTE physician. Overall, encompassing both patient and clinician phases of decision‐making, the average time from symptom onset to diagnosis in PSP was 3.26 (IQR = 2.42, 4.75) years and in CBS 2.58 (IQR = 1.69, 4.08) years (Table 3).

**TABLE 4:** Differential diagnoses and referral speciality in PSP and CBS

Frequency (percentage)	PSP, n = 52	CBS, n = 27
Frequency of alternative preceding diagnoses		
None	18 (34.6)	5 (18.5)
One	18 (34.6)	17 (63.0)
Multiple	11 (21.2)	2 (7.4)
Missing	5 (9.6)	5 (18.5)
Type/distribution of alternative preceding diagnoses^a^		
Parkinsonism	30 (57.7)	9 (33.3)
Unspecified parkinsonism	4 (13.3)	1 (11.1)
Parkinson's plus	2 (6.7)	0 (0.0)
PD	20 (66.7)	8 (88.8)
MSA	3 (10.0)	0 (0.0)
VP	1 (3.3)	0 (0.0)
Dementia	3 (5.8)	0 (0.0)
AD	1 (33.3)	0 (0.0)
FTD	1 (33.3)	0 (0.0)
Unspecified	1 (33.3)	0 (0.0)
Other	13 (25.0)	6 (22.2)
Vascular	3 (23.1)	1 (16.7)
Cord syndrome	2 (15.4)	0 (0.0)
Unspecified gait disorder	1 (7.7)	0 (0.0)
Spinocerebellar ataxia	1 (7.7)	0 (0.0)
Alcoholism	1 (7.7)	0 (0.0)
Age	1 (7.7)	0 (0.0)
Pesticide exposure	1 (7.7)	0 (0.0)
Depression/anxiety	1 (7.7)	2 (33.3)
Stress	1 (7.7)	0 (0.0)
Post viral fatigue	1 (7.7)	0 (0.0)
“Vertigo”	0 (0.0)	1 (16.7)
Functional neurological disorder	0 (0.0)	1 (16.7)
“Dyspraxia”	0 (0.0)	1 (16.7)
Speciality of secondary care referral		
Outpatient	26 (50.0%)	14 (51.9)
Neurology	9 (34.6)	6 (42.9)
COTE	5 (19.2)	1 (7.1)
Stroke	1 (3.8)	2 (14.3)
ENT	1 (3.8)	0 (0.0)
Ophthalmology	1 (3.8)	0 (0.0)
Unspecified	9 (34.6)	5 (35.7)
Inpatient	4 (7.7%)	0 (0.0%)
Neurosurgery	1 (25.0)	–
Stroke	1 (25.0)	–
General Medicine	1 (25.0)	–
COTE	1 (25.0)	–
Unsure	1 (1.9)	0 (0.0)
Thought never referred	1 (1.8)	0 (0.0)
Missing	20 (38.5)	13 (48.1)
Speciality giving final diagnosis of PSP/CBS		
Neurology	27 (51.9)	12 (44.4)
COTE	11 (21.2)	2 (7.4)
Neurosurgery	1 (1.9)	0 (0.0)
GP	1 (1.9)	0 (0.0)
Unspecified	1 (1.9)	2 (7.4)
Nurse	1 (1.9)	0 (0.0)
Missing	10 (19.2)	11 (40.7)

Abbreviations: AD, Alzheimer's disease; CBS, corticobasal syndrome; COTE, Care of the Elderly; ENT, ear nose and throat; FTD, frontotemporal dementia; GP, general practitioner; MSA, multiple system atrophy; PD, Parkinson's disease; PSP, progressive supranuclear palsy; VP, vascular parkinsonism.

^a^ Subcategories not mutually exclusive.

## Discussion

Early diagnosis is essential to advance the translation of future therapeutic approaches into successful disease‐modifying agents, as well as for clinical care. The starting point of an individual's journey toward diagnosis, as suggested by the Anderson model, is the detection of unexplained symptoms. Thereafter, individual help‐seeking behavior involves not only whether an individual seeks help for such symptoms, but also the timing of that decision. At symptom detection, fewer PSP patients compared to those with CBS immediately infer illness (a quarter of those with PSP vs. a third of those with CBS). In those not immediately evaluating their symptoms as serious, PSP patients also take three times longer (6 months on average) than those with CBS to ultimately infer illness. In both PSP and CBS, there is little subsequent illness or behavioral delay.

Differences in index symptoms identified by individuals with PSP and their caregivers, and the self‐reported inability of some patients to detect a departure from normality suggests that the time interval from symptom onset to symptom detection may also contribute to overall diagnostic delay. This time interval, unmeasured in the Anderson model, may be particularly relevant in neurodegenerative conditions such as PSP/CBS where disease‐related factors, including early cognitive or impaired insight, may influence symptom detection, which is a barrier previously identified in analyses exploring help‐seeking before a diagnosis of dementia.^7^ Thereafter, the lower specificity of index symptoms in PSP, and the lower prevalence of multiple index symptoms compared to CBS, might influence the initial perceived severity of index symptoms and the longer time to attribute such symptoms as serious or because of illness. Differences in appraisal duration may also reflect differences in the self‐monitoring strategies or mechanisms used by patients to appraise symptoms. Older people, for example, are known to normalize illness in the context of their age.^7^, ^8^ New or evolving somatic information may be masked by or attributed to co‐existing medical conditions or other contexts such as injury or medication.^9^ Information‐seeking as an appraisal strategy, including the use of social contacts such as family members, may also influence symptom interpretation and appraisal duration.^10^ None of these potential explanations readily explain the differences seen in PSP and CBS, however.

Unlike those with PSP and CBS, for whom there was little subsequent illness or behavioral delay having inferred illness, a previous study of individuals with PD in 11 European countries found that 50% of PD patients waited over 6 months (including 29% who waited 12 months or longer) from first noticing symptoms before seeking healthcare professional input. In the same study, those in rural areas waited longer to seek help than those in urban areas, with no difference due to age or sex.^11^ In our study, the nature of symptoms, in particular the extent to which experienced symptoms were associated with a risk of or an incident causing harm, as well as their functional impact, appear to have contributed to shorter symptom appraisal before illness inference. In more PSP patients compared to CBS patients (19.2% vs. 11.1%), the decision to seek medical attention was primarily because of the recommendation of family members, highlighting the importance of the caregiver role in influencing illness and/or behavioral delay, as well as appraisal delay. Individuals with a smaller social network, perhaps because of advancing age, may, therefore, be more vulnerable to diagnostic delay. In CBS, ~11% of patients sought input from their GP due to a concern their symptoms were because of another diagnosis, suggesting that the crystallization of symptoms into a particular diagnosis (even if inaccurate) may shorten illness delay. In PD, however, individuals have actively decided against seeking help because of a suspicion their symptoms may be because of PD.^12^ Of note in our study, illness inference, although inherent in the current definition of appraisal delay in the Anderson model, was not necessary for help‐seeking.  Several individuals, for example, ultimately concluded their symptoms were due to illness based on the actions of their GP, having first sought this input. In such contexts, clinician input before illness inference may instead be sought for symptomatic relief, as a strategy used in appraising symptoms or as an anxiety reducing tool. Previous studies in PD have also shown that an individual's decision to seek healthcare input is influenced by their perception of how their healthcare provider might perceive their presentation.^11^ Patient‐provider interactions, potentially determined by a complex context of previous successful or unsuccessful interactions and communication, likely also influence illness and behavioral delay, as well as patient and caregiver expectations about subsequent management after this initial contact.

Having reached primary care, the average duration between an individual's first attendance and their self‐reported first referral to secondary care was similar (3 months in PSP and 2 months in CBS). In addition to the potential negative consequences of referral delay, with respect to unscheduled emergency healthcare utilization (both out of hours primary care services as well as secondary care services), the perceived reluctance of some GPs to refer to secondary care resulted in additional appointments with another GP or the use of private healthcare. Although several initial GP referrals were sent to neurology or COTE (in keeping with NICE/SIGN guidelines),^13^, ^14^ a range of other speciality referrals were made, likely reflecting the non‐specificity of symptoms. PSP patients, in particular, reported multiple referrals and speciality reviews before diagnosis, as well as emergency admissions, further highlighting the complexity of the diagnostic journey in this patient group and the multiplicity of interactions between primary and secondary care before diagnosis. The breadth and extent of preceding diagnoses and investigations reported, likely reflecting clinician diagnostic uncertainty, will also impact treatment delay duration.

Overall, the average time from symptom onset to diagnosis was 3.26 (IQR = 2.42, 4.75) years in PSP and 2.58 (IQR = 1.69, 4.08) years in CBS. Time to diagnosis based on self‐report, nationally across the health‐boards of Scotland, is therefore, similar to time from index symptom onset to primary unchanging diagnosis (4.03 years [IQR = 2.31, 6.19]) reported in incident PSP/CBD patients recruited to the Parkinsonism Incidence in North‐East Scotland (PINE) study from a single health‐board, National Health Service (NHS) Grampian, between 2002 and 2009, where time intervals were based on medical record documentation rather than self‐report.^15^

The primary strength of this study is the detailed evaluation of the extent to which patient‐dependent phases of decision‐making contribute to overall diagnostic delay in PSP/CBS patients recruited nationally in Scotland across several NHS health‐boards, using the time intervals of an established theoretical framework. Our mixed‐method approach facilitates a greater understanding of the potential determinants of the duration of such phases of decision‐making, particularly those that could be modified to reduce diagnostic delay. As with all retrospective surveys, however, non‐response, reporting, or recall bias may influence results. A key limitation of this study is our current inability to corroborate self‐report with an authoritative source, such as the primary and secondary care medical record, although we have consent to review participants medical records after death so we may be able to evaluate this further in the future. The extent of motor or cognitive disease severity, and whether individuals met relevant diagnostic criteria when they first presented to primary or secondary care, has also not been evaluated. Sample size is also small, although this in keeping with many studies in PSP/CBS because of the rarity of these diseases, as well as mixed‐method analyses.

In conclusion, although patient‐dependent appraisal delay contributes to overall diagnostic delay in PSP/CBS, the greater proportion of overall diagnostic delay, from symptom detection to diagnosis, arises after contact with a healthcare professional. This contrasts with cancer diagnoses where, when GPs and health systems are more vigilant and aware of these diagnoses, a key public health challenge arises from reducing delays in patient symptom appraisal and GP help‐seeking. The Anderson model, although a useful framework, rather oversimplifies the complex and frequently non‐linear diagnostic period after an initial healthcare professional contact (treatment delay), especially in rare, neurodegenerative diseases. In this period, for example, there are often several phases to diagnostic delay, including the transition between primary and secondary care and the time to identification of syndromic (eg, parkinsonism) versus specific (eg, PSP) diagnoses.^15^ Treatment commencement within the Anderson Model as a diagnostic milestone is also open to variation in interpretation. Further conceptualization and expansion of the treatment delay phase is, therefore, necessary to allow standardized comparisons between studies and maximize its use as a standardized framework in diagnostics research in neurodegenerative disease. Quantitative analyses to verify and compare the magnitude of the effect of identified variables that influence help‐seeking behavior will also be required to develop effective interventions that reduce delay at all stages of the diagnostic journey in PSP/CBS.

## Author Roles

(1) Research project: A. Conception, B. Organization, C. Execution; (2) Statistical Analysis: A. Design, B. Execution, C. Review and Critique; (3) Manuscript: A. Writing of the First Draft, B. Review and Critique.

D.M.A.S.: 1A, 1B, 1C, 2A, 2B, 3A, 3B

P.M.: 1A, 3B

C.E.C.: 1A, 1B, 1C, 2A, 2C, 3B

## Disclosures

**Ethical compliance statement:** The study was approved by the NHS Grampian Research Ethics Committee and the Multicentre Research Ethics Committee A for Scotland. Written informed consent was obtained from all participants or guardians of participants with incapacity. We confirm that we have read the Journal's position on issues involved in ethical publication and affirm that this work is consistent with those guidelines.

**Funding sources and Conflicts of interest:** Chief Scientist Office (CSO) of the Scottish Government, PSP Association. The authors declare that there are no conflicts of interest of interest relevant to this work.

**Financial Disclosures for the previous 12 months:** The authors declare that there are no additional disclosures to report.

## Supporting information


**Table S1.** Baseline and 6‐month clinical assessments in PSP/CBS participants.
**Table S2.** PSP/CBS patient and caregiver questions on diagnostic pathway.
**Table S3.** Baseline and 6‐month clinical assessments in PSP/CBS caregivers.
**Table S4.** Baseline clinical assessments in age‐, sex‐ and disease duration matched PD participants.
**Table S5.** Decision making (appraisal delay, illness delay and behavioral delay) in individuals with progressive supranuclear palsy and corticobasal syndrome.
