# Supplementary material for: Total Patient Delay: A Comparison of Patient and Clinician/Health System Delays in the Diagnosis of Progressive Supranuclear Palsy and Corticobasal Syndrome
Source: Mov Disord Clin Pract. 2024 Feb 18;11(5):478–87. doi: 10.1002/mdc3.13990 (PMC11078481; doi:10.1002/mdc3.13990)
Supplement: Supplementary file 1 — Table S1. Baseline and 6‐month clinical assessments in PSP/CBS participants. Table S2. PSP/CBS patient and caregiver questions on diagnostic pathway. Table S3. Baseline and 6‐month clinical assessments in PSP/CBS caregivers. Table S4. Baseline clinical assessments in age‐, sex‐ and disease duration matched PD participants. Table S5. Decision making (appraisal delay, illness delay and behavioral delay) in individuals with progressive supranuclear palsy and corticobasal syndrome. [file MDC3-11-478-s001.docx]

**Supplementary Table 1:** Baseline and 6-month clinical assessments in PSP/CBS participants

| **PSP/CBS Patients** | **Case Item** |
| --- | --- |
| **Study Visit 0**  **(0 months)** | Registration and informed consent |
|  | Demographics |
|  | Patient history/symptom review |
|  | Medical history/Medication |
|  | Neurological Examination and Movement Disorder Society Unified Parkinson Disease Rating Scale Part 3 (MDS-UPDRS 3) (videoed)^1^ |
|  | Edinburgh Motor Assessment Scale (EMAS)^2^ |
|  | Addenbrookes Cognitive Examination-III (ACE-III)^3^ |
|  | Edinburgh Cognitive Assessment Scale (ECAS)^4^ |
|  | Multi-domain PSP Rating Scale^5^ |
|  | Schwab & England^6^ |
|  | Barthel Index^7^ |
|  | Euroquol EQ5D^8^ |
|  | World Health Organisation Disability Scale 2.0^9^ |
|  | PSP Quality of Life scale^10^ |
|  | Parkinson’s Disease Questionnaire-8 (PDQ-8)^11^ |
|  | Hospital Anxiety and Depression Scale (HADS)^12^ |
|  | Diagnostic pathways (see Appendix 3) |
|  | Healthcare interventions (see Appendix 3) |
|  | Activities of daily living (see Appendix 3) |
|  | **Total** |
| **Postal Assessment 1**  **(6 months)** | Patient history/symptom review |
|  | Medical history/Medication |
|  | Schwab & England |
|  | Barthel Index |
|  | Euroquol EQ5D |
|  | World Health Organisation Disability Scale 2.0 |
|  | PSP Quality of Life scale |
|  | PDQ-8 |
|  | HADS |
|  | Healthcare interventions |
|  | Activities of daily living |
|  | **Total** |

ACE-III=Addenbrookes Cognitive Examination-III, ECAS=Edinburgh Cognitive Assessment Scale, EMAS Edinburgh Motor Assessment Scale, HADS= Hospital Anxiety and Depression Scale, MDS-UPDRS3=Movement Disorder Society Unified Parkinson Disease Rating Scale Part 3, PDQ-8=Parkinson’s Disease Questionnaire-8, PSP=progressive supranuclear palsy.

**Supplementary Table 2:** PSP/CBS patient and caregiver questions on diagnostic pathway

| **Patient questions diagnostic pathways** |
| --- |
| What were the first symptoms you experienced which turned out to be related to PSP or CBD? |
| Did you think your symptoms were serious, or due to an illness, when they first started? |
| If not, what did you think the symptoms were due to? |
| Roughly how long after your symptoms started, did you begin to think they might be due to a significant illness? What factors made you come to this conclusion? |
| How long after your first symptoms started did you decide to see a doctor e.g. your GP? |
| What factors influenced your decision to see a doctor? |
| Once you had made a decision to see your doctor, did you delay or hold off in making an appointment? If yes, did anything in particular influence this delay? |
| When did you first see your GP about your symptoms (month/year)? |
| When (month/year), and to which hospital speciality did your GP refer you to investigate your symptoms? |
| What investigations did you have prior to your diagnosis? |
| When (month/year), and from whom, did you receive your diagnosis of PSP or CBD? |
| At any stage did you receive another explanation for your symptoms other than PSP or CBD? If yes, from whom, and what explanation or other diagnosis were you given? |
| **Caregiver questions diagnostic pathways** |
| What were the first symptoms you noticed relating to your relative’s diagnosis of PSP or CBD? |
| Roughly how long after their first symptoms started did your relative decide to see a doctor e.g. GP? |
| When (month/year), and from whom, did your relative receive their diagnosis of PSP or CBD? |
| At any stage did they receive another explanation for their symptoms other than PSP or CBD? If yes, from whom, and what explanation were they given? |
| What investigations did your relative have prior to their diagnosis? |

**Supplementary Table 3** Baseline and 6-month clinical assessments in PSP/CBS caregivers

| **Carer** | **Carer Item** |
| --- | --- |
| **Study visit 0**  **(0 months)** | Registration and informed consent |
|  | Demographics and caring input |
|  | Medical history/Medication |
|  | Zarit burden questionnaire^13^ |
|  | Euroquol EQ5D |
|  | HADS |
|  | ECAS Carer screen |
|  | Frontotemporal dementia functional rating scale (FTD-FRS) (relating to patient)^14^ |
|  | Neuropsychiatric inventory (relating to patient)^15^ |
|  | Barthel Index -proxy^*^ |
|  | World Health Organisation Disability Scale 2.0 -proxy* |
|  | PSP Quality of Life Scale -proxy^*^ |
|  | Diagnostic pathways-proxy^*^ |
|  | Healthcare interventions -proxy^*^ |
|  | Activities of daily living - proxy^*^ |
|  | **Total** |
| **Postal assessment 1**  **(6 months)** | Medical history/Medication |
|  | Caring input |
|  | Zarit burden questionnaire |
|  | Euroquol EQ5D |
|  | HADS |
|  | ECAS Carer screen |
|  | FTD-FRS (relating to patient) |
|  | Neuropsychiatric inventory (relating to patient) |
|  | Barthel Index - proxy^*^ |
|  | World Health Organisation Disability Scale 2.0 -proxy^*^ |
|  | PSP Quality of Life Scale-proxy^*^ |
|  | Healthcare interventions -proxy^*^ |
|  | Activities of daily living - proxy^*^ |
|  | **Total** |

^*^Proxy = caregiver rating of PSP/CBS patient scales. ECAS=Edinburgh Cognitive Assessment Scale, FTD-FRS=Frontotemporal dementia functional rating scale, HADS= Hospital Anxiety and Depression Scale, PSP=progressive supranuclear palsy.

**Supplementary Table 4:** Baseline clinical assessments in age-, sex- and disease duration matched PD participants

| **PD Patient** | **PD Case item** |
| --- | --- |
|  | Consent |
|  | *MDS-UPDRS 3 (videoed) |
|  | *EMAS |
|  | *ACE-III |
|  | *ECAS |
|  | *Multi-domain PSP Rating Scale |
|  | **Total** |

ACE-III=Addenbrookes Cognitive Examination-III, ECAS=Edinburgh Cognitive Assessment Scale, EMAS Edinburgh Motor Assessment Scale, MDS-UPDRS3=Movement Disorder Society Unified Parkinson Disease Rating Scale Part 3, PSP=progressive supranuclear palsy.

**Supplementary Table 5:** Decision making (appraisal delay, illness delay and behavioural delay) in individuals with progressive supranuclear palsy and corticobasal syndrome

|  | **Progressive supranuclear palsy**  **n=52** | **Frequency (percentage)^*^** | **Corticobasal syndrome**  **n=27** | **Frequency (percentage)** |
| --- | --- | --- | --- | --- |
| **Appraisal delay** | **Serious illness inferred at symptom onset**  Nature, persistence or perceived consequences of symptoms e.g.falls injury  Misattribution of symptoms to other diagnoses (PD or MS)  Actions/perceived concern of GP (reason for contact with GP unclear)  Unable to recall reasons | **13 (25.0)**  8 (61.5)  3 (23.1)  1 (7.7)  1 (7.7) | **Serious illness inferred at symptom onset**  Nature, consequences (hospitalisation due to a fall) or progression of symptoms  Misattribution of symptoms to other diagnoses  Due to referral to secondary care | **9 (33.3)**  5 (55.6)  3 (33.3)  1 (11.1) |
|  | **Serious illness not inferred at symptom onset**  Uncertain of symptom cause, but did not immediately infer illness  Normal physiological variation (e.g., age, deteriorating vision, clumsiness)  Other neurological diagnoses self-labelled as non-serious  Stress/anxiety  Exogenous: medication changes or recovery from a recent accident  Self-rated symptoms as trivial  Did not recognise departure from normality  **Serious illness eventually inferred**  Specific symptoms (including safety concerns or lack of improvement)  Only inferred at the time of diagnosis  Unable to recall reasons  Actions of others (family concern, GP referral to secondary care)  Attribution of symptoms to PD  Unspecified health anxiety  Observation of similar symptoms in a diagnosed friend | **22 (42.3)**  7 (31.8)  6 (27.3)  3 (13.6)  2 (9.1)  2 (9.1)  2 (9.1)  2 (9.1)  **22 (42.3)**  9 (17.3)  5 (22.7)  3 (13.6)  3 (13.6)  2 (9.1)  1 (4.5)  1 (4.5) | **Serious illness not inferred at symptom onset**  Normal physiological variation (age, clumsiness, lack of use, fatigue)  Musculoskeletal symptoms or “trapped nerve”  Identified, uncertain of symptom cause, but did not immediately infer illness  **Serious illness eventually inferred**  Progression or lack of improvement  Due to referral to secondary care  Belief symptoms were due to injury  Functional impairment (driving)  Unspecified health anxiety | **7 (25.9)**  4 (57.1)  2 (28.6)  1 (14.3)  **7 (25.9)**  3 (42.9)  1 (14.3)  1 (14.3)  1 (14.3)  1 (14.3) |
|  | Total  Missing data  Incapacity | 35 (67.3)  17 (32.7)  12 (70.6) | Total  Missing data  Incapacity | 16 (59.3)  11 (40.7)  9 (81.8) |
| **Illness delay** | Due to nature of experienced symptoms  Wish or recommendation of others (family or work colleagues)  No improvement in symptoms & an explanation from a GP therefore felt necessary  Functional difficulties (struggling at work, difficulty getting in and out of a car, recurrent falls off bike)  Belief that medication changes were required  Unable to recall  Specific diagnosis suspected (MS)  Multiple ED attendances  Referred by a secondary care physician at an unrelated appointment | 10 (19.2)  10 (19.2)  4 (7.7)  3 (5.8)  2 (3.8)  2 (3.8)  1 (1.9)  1 (1.9)  1 (1.9) | Due to nature of experienced symptoms  Due to the concern of their family  Concern about another diagnosis  Having researched their symptoms  Unspecified health anxiety  Did not decide to see their GP: referred from secondary care or emergency admission | 6 (22.2)  3 (11.1)  3 (11.1)  1 (3.7)  1 (3.7)  2 (7.4) |
|  | Total  Missing data  Incapacity | 34 (65.4)  18 (34.6)  12 (66.7) | Total  Missing data  Incapacity | 16 (59.3)  11 (40.7)  9 (81.8) |
| **Behavioural delay** | No delay  Concluded symptoms due to medication therefore no urgency  Waited for another reason to attend their GP  No specific reason given  Insight into the possibility of receiving a diagnosis they wished to defer | 30 (57.7)  1 (1.9)  1 (1.9)  1 (1.9)  2 (3.8) | No delay  Persistent hope that symptoms would resolve | 13 (48.1)  1 (3.7) |
|  | Total  Missing data  Incapacity | 35 (67.3)  17 (32.7)  12 (70.6) | **Total**  Missing data  Incapacity | 14 (51.9)  13 (48.1)  9 (69.2) |

^*^subcategories not mutually exclusive. ED= Emergency Department; GP= General Practitioner; MND=motor neuron disease; MS=multiple sclerosis PD=Parkinson’s disease.

Supplementary Table References

1. Martinez-Martin P, Rodriguez-Blazquez C, Alvarez-Sanchez M, et al. Expanded and independent validation of the Movement Disorder Society–Unified Parkinson’s Disease Rating Scale (MDS-UPDRS). *Journal of Neurology.* 2013;260(1):228-236.

2. Elamin M, Bennett G, Symonds A, et al. Introducing a Brief Screening a Tool for Motor Signs in Patients with Dementia (P6.203). *Neurology.* 2015;84(14 Supplement):P6.203.

3. Hsieh S, Schubert S, Hoon C, Mioshi E, Hodges JR. Validation of the Addenbrooke's Cognitive Examination III in Frontotemporal Dementia and Alzheimer's Disease. *Dementia and Geriatric Cognitive Disorders.* 2013;36(3-4):242-250.

4. Niven E, Newton J, Foley J, et al. Validation of the Edinburgh Cognitive and Behavioural Amyotrophic Lateral Sclerosis Screen (ECAS): A cognitive tool for motor disorders. *Amyotrophic Lateral Sclerosis and Frontotemporal Degeneration.* 2015;16(3-4):172-179.

5. Golbe LI, Ohman-Strickland PA. A clinical rating scale for progressive supranuclear palsy. *Brain.* 2007;130(Pt 6):1552-1565.

6. Schwab J. F. EAC. Projection technique for evaluating surgery in Parkinson's disease. In: Gillingham F. J. DMC, ed. *Proceedings of the Third Symposium on Parkinson's Disease.* Edinburgh, UK: Churchill Livingstone; 1969:152-157.

7. Wade DT, Collin C. The Barthel ADL Index: a standard measure of physical disability? *Int Disabil Stud.* 1988;10(2):64-67.

8. Siderowf AD, Werner RM. The EQ-5D--a generic quality of life measure--is a useful instrument to measure quality of life in patients with Parkinson's disease. *J Neurol Neurosurg Psychiatry.* 2001;70(6):817.

9. Rehm J, Üstün TB, Saxena S, et al. On the development and psychometric testing of the WHO screening instrument to assess disablement in the general population. *International Journal of Methods in Psychiatric Research.* 1999;8(2):110-122.

10. Schrag A, Selai C, Quinn N, et al. Measuring quality of life in PSP: the PSP-QoL. *Neurology.* 2006;67(1):39-44.

11. Jenkinson C, Fitzpatrick R, Peto V, Greenhall R, Hyman N. The PDQ-8: Development and validation of a short-form parkinson's disease questionnaire. *Psychology & Health.* 1997;12(6):805-814.

12. Zigmond AS, Snaith RP. The hospital anxiety and depression scale. *Acta Psychiatr Scand.* 1983;67(6):361-370.

13. Bédard M, Molloy DW, Squire L, Dubois S, Lever JA, O'Donnell M. The Zarit Burden Interview: a new short version and screening version. *Gerontologist.* 2001;41(5):652-657.

14. Mioshi E, Hsieh S, Savage S, Hornberger M, Hodges JR. Clinical staging and disease progression in frontotemporal dementia. *Neurology.* 2010;74(20):1591-1597.

15. Cummings JL, Mega M, Gray K, Rosenberg-Thompson S, Carusi DA, Gornbein J. The Neuropsychiatric Inventory: comprehensive assessment of psychopathology in dementia. *Neurology.* 1994;44(12):2308-2314.
